# Needle‑based gastrocnemius lengthening: a novel ultrasound‑guided noninvasive technique: part II—clinical results

**DOI:** 10.1186/s13018-024-04685-0

**Published:** 2024-03-26

**Authors:** A. Iborra, M. Villanueva, H. Fahandezh-Saddi Díaz

**Affiliations:** 1 Institute Avanfi, 28020 Madrid, Spain; 2 Unit for Ultrasound‑Guided Surgery, Hospital Beata Maria Ana, Madrid, Spain; 3 Department of Podiatry, Faculty of Health Sciences, University of La Salle Madrid, Madrid, Spain; 4 Unit of Foot and Ankle Surgery, Hospital La Zarzuela, Madrid, Spain

**Keywords:** Equinus foot, Gastrocnemius recession, Ultrasound-guided surgery, Gastrocnemius tendon lengthening

## Abstract

**Background:**

Isolated gastrocnemius contracture has been associated with more than 30 lower limb disorders, including plantar heel pain/plantar fasciitis, Achilles tendinosis, equinus foot, adult flatfoot, and metatarsalgia. Although many techniques are available for gastrocnemius recession, potential anesthetic, cosmetic, and wound-related complications can lead to patient dissatisfaction. Open and endoscopic recession techniques usually require epidural or general anesthesia, exsanguination of the lower extremities and stitches and can damage the sural nerve, which is not under the complete control of the surgeon at all stages of the procedure. The purpose of this study is to evaluate the clinical results of a surgical technique for gastrocnemius lengthening with a needle, as previously described in cadaver specimens.

**Methods and results:**

We performed a prospective study of ultrasound-guided gastrocnemius tendon lengthening in level II using a needle in 24 cases (19 patients) of gastrocnemius contracture. The study population comprised 12 males and 7 females. Mean age was 41 years (18–64). All but 5 recessions were bilateral and occurred simultaneously. The indication for the procedure was gastrocnemius contracture; although the patients also presented other conditions such as non-insertional Achilles tendinopathy in 6 patients (2 were bilateral), insertional Achilles calcifying enthesitis in 4 (1 was bilateral), metatarsalgia in 4, flexible flat foot in 1 and plantar fasciitis in 5 (2 were bilateral). The inclusion criteria were the failure of a previous conservative protocol, that the Silfverskiöld test was positive, and that the pathology suffered by the patient was within the indications for surgical lengthening of the patients and were described in the scientific literature. The exclusion criteria were that the inclusion criteria were not met, and patients with surgical risk ASA 3 or more and children. In these patients, although possible, it is preferable to perform the procedure in the operating room with monitoring, as well as in children since they could be agitated during the procedure at the office. We used the beveled tip of an Abbocath needle as a surgical scalpel. All patients underwent recession of the gastrocnemius tendon, as in an incomplete Strayer release. We evaluated pre- and postoperative dorsiflexion, outcomes, and procedural pain (based on a visual analog scale and the American Orthopedic Foot and Ankle Society scores), as well as potential complications. No damage was done to the sural bundle.

**Results:**

Ankle dorsiflexion increased on average by 17.89°. The average postoperative visual analog score for pain before surgery was 5.78, 5.53 in the first week, 1.89 at 1 month, and 0.26 at 3 months, decreasing to 0.11 at 9 months. The mean postoperative American Orthopedic Foot and Ankle Society Ankle-Hindfoot score the average was 50.52 before surgery, 43.42 at 1 week, 72.37 at 1 month, 87.37 at 3 months, and 90.79 at 9 months.

**Conclusion:**

Ultrasound-guided needle lengthening of the gastrocnemius tendon is a novel, safe, and effective technique that enables the surgeon to check all the structures clearly, thus minimizing the risk of neurovascular damage. The results are encouraging, and the advantages of this approach include absence of a wound and no need for stitches. Recovery is fast and relatively painless. A specific advantage of ultrasound-guided needle lengthening of the gastrocnemius tendon is the fact that it can be performed in a specialist's office, with a very basic instrument set and local anesthesia, thus reducing expenses.

## Introduction

Gastrocnemius contracture is defined as ankle dorsiflexion < 10° with the knee extended. Barouk et al. [1] described the Silfverskiöld maneuver as the appropriate technique for differentiating gastrocnemius contracture from gastrocnemius-soleus contracture. DiGiovanni et al. recommended a definition based on their study: ankle joint dorsiflexion < 5° with the knee extended for gastrocnemius equinus and < 10° with the knee flexed for gastrocnemius-soleus equinus is considered abnormal [2]. The amount of dorsiflexion required for normal gait has been shown to be between 5° and 10° [3–5].

Isolated gastrocnemius contracture is thought to predispose to or aggravate numerous foot and ankle conditions such as plantar fasciitis, Achilles tendinosis, flatfoot, clubfoot, diabetic foot ulcer, knee hyperextension (genu recurvatum), metatarsalgia, midfoot pain or arthritis, lateral foot pain, and nerve entrapment. In children, the deformity has been associated with equinus, spasticity, and cerebral palsy [6–17].

Although isolated gastrocnemius recession was described more than a century ago [18], reports of this technique being used specifically as a primary intervention to treat foot pain in adults have only recently begun to emerge. Cychosz et al. [19] performed the first systematic review to assign grades of recommendation to gastrocnemius recession as a therapeutic intervention for the indications listed above. More studies are required to establish the true efficacy and indications for gastrocnemius lengthening.

Gastrocnemius recession can be performed proximally or distally as open surgery, as an endoscopic procedure, or as ultrasound-guided surgery [1, 15]. Open techniques may lead to wound dehiscence, infections, and poor cosmetic results. The endoscopic technique is associated with fewer complications than open surgery but still requires exanguination of the leg [20, 21], and both techniques have been associated with neurovascular complications, such as injury of the sural nerve.

Ultrasound-guided gastrocnemius resection was first described in 2016 by Villanueva et al [15]. The technique consists [22, 23] of sectioning the gastrocnemius tendon through anatomical level II, as in the Strayer technique, with a surgical hook-knife. In 2019, the same authors described proximal resection of the medial head of the calf muscle [24].

Unlike open and endoscopic techniques, ultrasound-guided gastrocnemius recession is performed without exsanguination and with local anesthesia plus sedation and does not require stitches. The potential benefits include shorter recovery time, fewer complications, reduced morbidity, and the possibility of performing bilateral procedures [15, 24].

In 2022, Iborra et al. [25]. Described the ultrasound-guided gastrocnemius recession technique using a needle in cadaver specimens. The same authors had previously described ultrasound-guided surgical techniques with a needle, namely, plantar fasciotomy and aponeurotomy for Dupuytren’s disease [26, 27]. The use of an Abbocath needle for ultrasound-guided surgery has also been described for carpal tunnel syndrome [28] based on the same concept.

The aim of this study was to evaluate the results and complications of ultrasound-guided recession of the gastrocnemius tendon at level II using an Abbocath 16G needle.

## Material and methods

Our prospective study was performed in accordance with the principles of the 1964 Declaration of Helsinki (2013 revision) and approved by the Research Ethics Committee of Hospital Beata María. All participants gave their informed consent to participate in the study and for their clinical and radiological data to be reproduced. All authors contributed to patient selection, procedure and application of scores.

Between June 2022 and February 2023, we performed ultrasound-guided gastrocnemius tendon lengthening using a needle at level II in 24 cases (19 patients).

The study population comprised 12 males and 7 females. Mean age was 41 years (18–64). All but 5 recessions were bilateral and occurred simultaneously. The indication for the procedure was gastrocnemius contracture; although the patients also presented other conditions such as non-insertional Achilles tendinopathy in 6 patients (2 were bilateral), insertional Achilles calcifying enthesitis in 4 (1 was bilateral), metatarsalgia in 4, flexible flat foot in 1 and plantar fasciitis in 5 (2 were bilateral).

The zone 2 extends from the end of the medial belly of the gastrocnemius muscle to the end of the soleus muscle. This zone is characterized by condensation of the gastrocnemius fascia into a broad aponeurosis, which lies superficial to the soleus fascia. The patients had foot conditions such as Achilles tendonitis, calcifying enthesis, flatfoot, metatarsalgia, and plantar fasciitis. Gastrocnemius tension was assessed clinically using the Silfverskiöld test.

Pain was assessed using a visual analog scale (VAS, from 0 [no pain] to 10 [severe pain]) at baseline, 1 week, 1 month, 3 months, and 9 months after surgery.

Pain funtion and alignment was also assessed using the American Orthopedic Foot and Ankle Society (AOFAS) Ankle-Hindfoot score (pain, 40 points; function, 50 points; alignment, 10 points) at baseline, 1 week, 1 month, 3 months, and 9 months after surgery.

The inclusion criteria were the failure of a previous conservative protocol, that the Silfverskiöld test was positive, and that the pathology suffered by the patient was within the indications for surgical lengthening of the gastrocnemius tendon and were described in the scientific literature. The exclusion criteria were that the inclusion criteria were not met, and patients with surgical risk ASA 3 or more and children. ASA 3 patients were excluded from the study since in these patients, due to their health status, it is preferable to perform the procedure in the operating room with monitoring, as well as children since they could be agitated during the procedure.

**Fig. 1:**
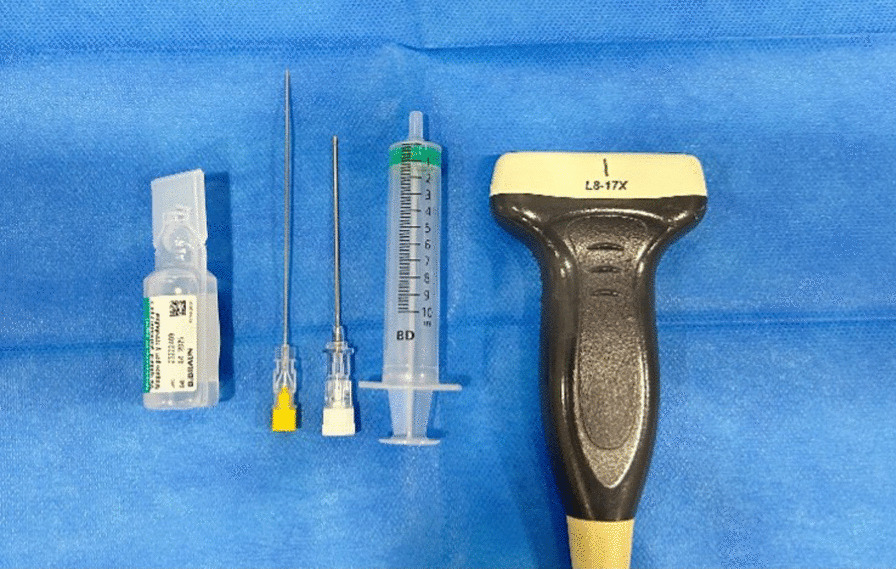
Instrument set: L8-17X multifrequency linear transducer (Alpinion Medical Systems, Bothell, WA, USA), 20G spinal needle, and Abbocath 16G needle

The instrument set included a 20G spinal needle, an Abbocath 16G needle, an ultrasound device (Alpinion ECube15) with a 10- to 17-MHz linear transducer, and the Needle Vision Plus™ software package. We used betadine gel as the ultrasound-gel in order to provide additional antiseptic measures (Fig. 1).

### Surgical technique

The surgical technique has been described elsewhere [25].

The patient was placed prone on the operating table, and the surgical field (from the popliteal fossa to the foot) was sterilized with povidone-iodine or chlorhexidine.

Ultrasound images in the transverse plane were used to delineate the anatomic boundaries of the gastrocnemius, Achilles tendon, soleus muscle, sural nerve, and sural vein.

The point for recession of the tendon was chosen within level II, usually 2 to 5 cm distal to the distal end of the medial gastrocnemius belly.

**Fig. 2:**
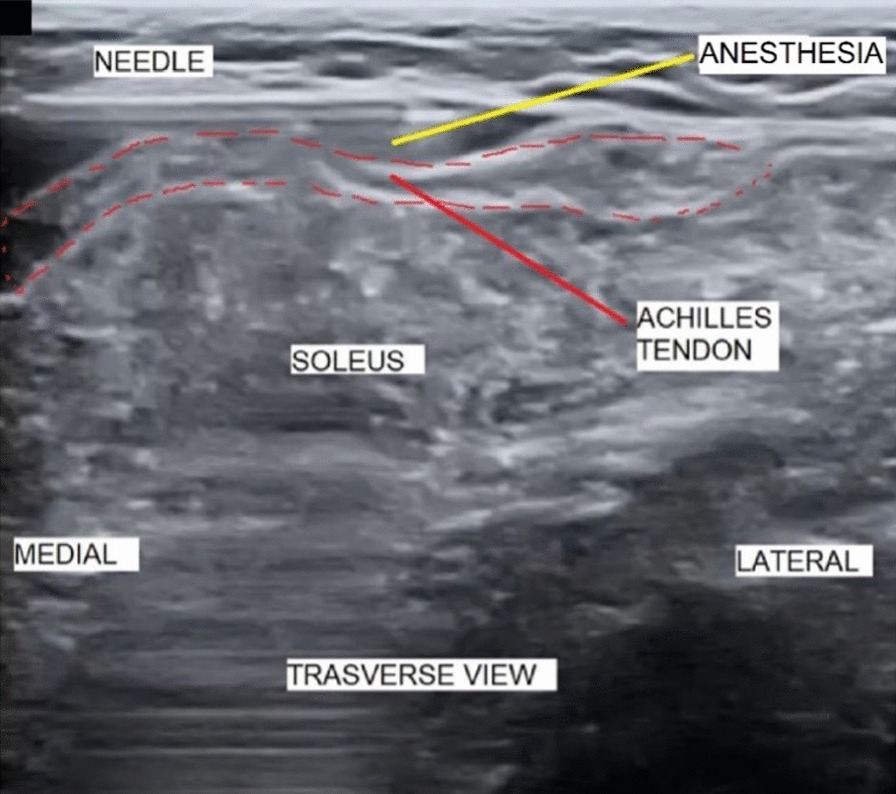
Injecting anesthesia between the Achilles tendon gastrocnemius and the fascia

With the transducer in the short or transverse axis, the nerve and sural vessels are always visible. At the chosen entry point, we inserted the 20G spinal needle from medial to lateral with both the transducer and the needle used to inject the local anesthesia transverse to the gastrocnemius tendon. We injected 2% mepivacaine (10–15 ml) into the superficial tendon-fascia and tendon-soleus muscle plane and waited 10 min before starting the resection. The needle was visible at all times (Fig. 2).

**Fig. 3:**
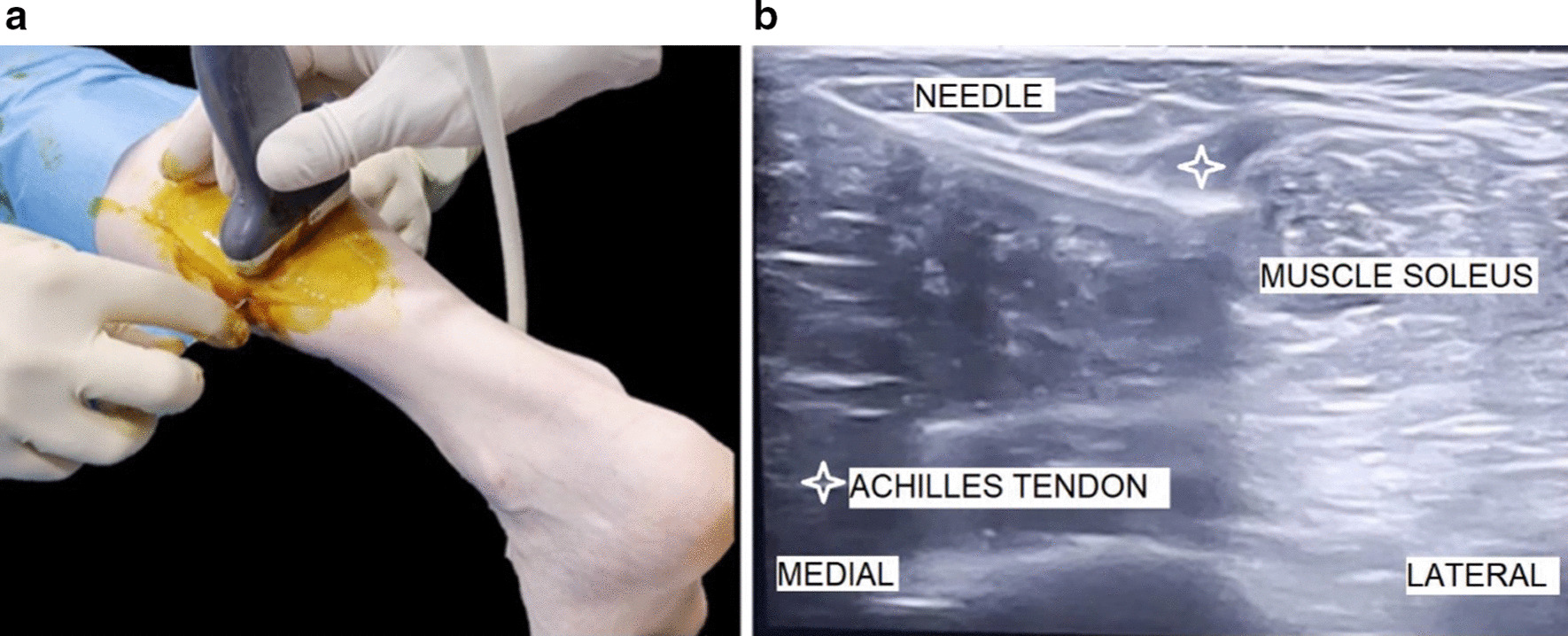
**a**, **b** Cutting the Achilles tendon of the gastrocnemius with a levering movement of the needle

With the ultrasound in a transverse position, the Abbocath needle was introduced through the same point where the anesthesia was injected and used to cut the gastrocnemius tendon with a levering movement, from superficial to deep, from deep to superficial, and from medial to lateral. It is important to remain in the same plane to make a single linear cut. The Abbocath bevel acts as a knife on the gastrocnemius tendon, thus enabling rapid and controlled progression of the recession (Fig. 3a,b).

During the cutting movement, it is very important for the assistant to keep the knee in extension and the ankle in forced dorsiflexion to create tension in the tendon and, therefore, render the release effective.

The transverse ultrasound plane must be maintained to achieve a continuous cutting line and thus prevent any tendon fibers from remaining uncut.

The resection was partial in all patients since the medial release was sufficient to achieve optimal lengthening of the gastrocnemius, although, if necessary, the surgeon could perform a complete resection, including the lateral part of the gastrocnemius tendon. We used only 1 entry point to ensure that the Abbocath needle was sufficiently long to cut the medial part of the tendon. The surgeon can control the progression of the recession until the desired gain in dorsiflexion is achieved.

The sural vein and nerve are usually located in the lateral and central area of the leg, making it very difficult to damage them in a partial resection of the medial part of the tendon. If the sural neurovascular bundle is located more medially and we need to advance the recession underneath it to achieve appropriate ankle dorsiflexion, we can inject more serum or anesthesia in order to separate and elevate the bundle and create a working space between the aponeurosis and the Achilles tendon of the gastrocnemius, an amount of 20–30 cc would be sufficient and safe to separate the neurovascular bundle, avoiding a possible compartment syndrome due to large volumes of serum as reported for our original technique with the hook-knife [15, 29]. In addition, we try to make the levering and cutting movement from superficial to deep, thereby avoiding uncontrolled damage of these structures with the tip of the Abbocath needle.

At the end of the procedure, the release is checked. The needle is moved from superficial to deep and vice-versa to ensure that no fibers remain uncut. Moreover, the process can be visualized directly by ultrasound in both the transverse and the longitudinal planes. After completion of the release, we inject 1 ml of bupivacaine 0.5% and 1 ml of betamethasone sodium phosphate (Celestone cronodose®) to reduce inflammation and pain. A small adhesive dressing is then placed over the needle entry point and a pressure bandage is placed, thus ending the procedure. The patient leaves the clinic walking, with partial weight bearing. Paracetamol is prescribed 1 g/8 h for 3–4 days, low-molecular weight heparin and antibiotic therapy were not required. Active dorsiflexion and plantar flexion of the ankle with the knee in extension are encouraged immediately after surgery and maintained for the first month.

Ankle dorsiflexion was measured while the clinician held the patient's foot in a neutral pronation-supination position and with the knee extended. All intraoperative measurements for each patient were obtained by the same author to standardize measurement. Ankle dorsiflexion was measured prior to surgery and after recession of the gastrocnemius tendon and at 3 and 9 months post-surgery.

**Fig. 4:**
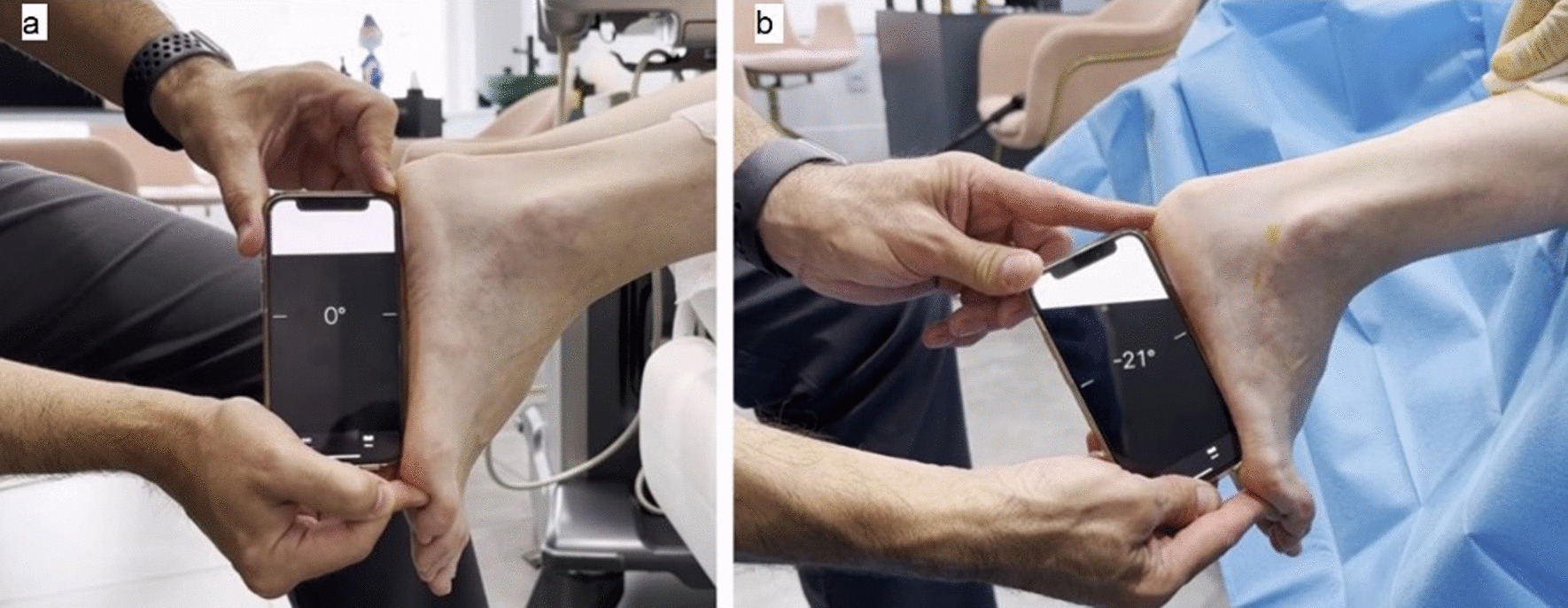
**a** and** b** Testing the range of motion of the ankle, before and after surgery. The statistical analysis was performed using SPSS 28.0 for Windows, and statistical significance was set at α < 0.05 based on a 2-tailed test.

We tested the range of motion of the ankle immediately after surgery (Fig. 4a, b).

## Results

**Fig. 5:**
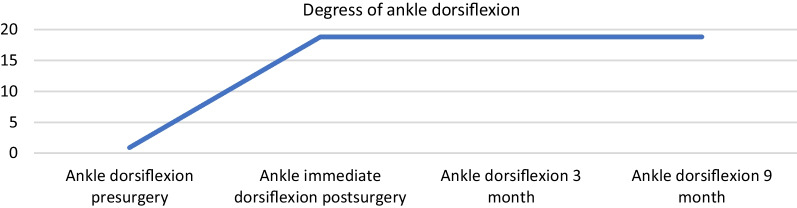
Progression degress of ankle dorsiflexion over time

A total of 24 limbs in 19 patients were included in the statistical analyses. In the clinical series, presurgical ankle dorsiflexion (mean 0.89°; standard deviation ± 1.56°) increased in all patients immediately after the surgical procedure (mean 18.79°; standard deviation ± 2.02°) and remained unchanged throughout the follow-up (3 and 9 months). The differences in ankle dorsiflexion measurements were statistically significant in each case (*p* < 0.001) (Fig. 5).

**Fig. 6:**
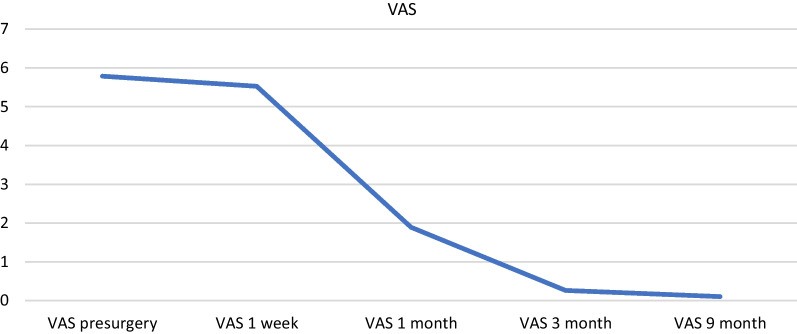
Progression of VAS over time

Postsurgical pain when walking was evaluated in the first week, mainly in the propulsion phase, which was described by all patients as very tolerable. Pain was managed during the first week with conventional analgesics and was evaluated using the VAS. The mean (SD) VAS value of pain before surgery was 5.78 (1,084), 5.52 (1.54) one week after surgery, 1.89 (1.1) at 1 month, and 0.26 at 3 months. Values remained largely unchanged at 9 months. The presurgery VAS score refers to the pain of the pathology, not the surgical pain (Fig. 6).

**Fig. 7:**
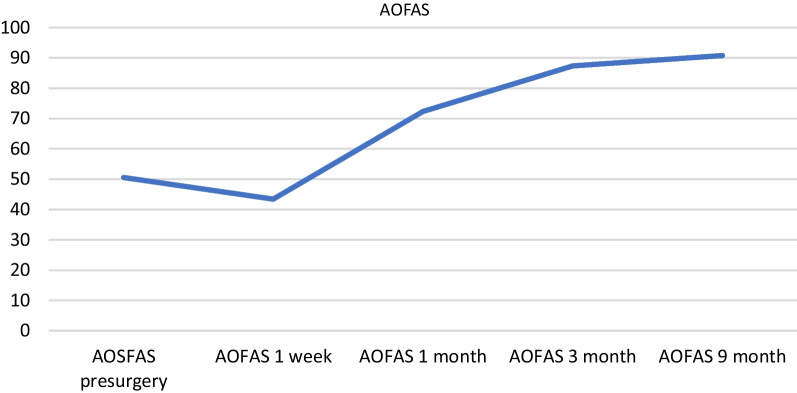
Progression of AOFAS over time

The postsurgical American Orthopedic Foot and Ankle Society Ankle-Hindfoot score remained stable since the third month after surgery. The average was 50.52 (range of 30–60) before surgery, 43.42 (range 20–60) at 1 week, 72.37 (range 50–85) at 1 month, 87.37 (range 80–100) at 3 months, and 90.79 (range 80–100) at 9 months. Pain and function improved significantly in all patients (Fig. 7).

We assessed statistical differences between preoperative and postoperative functional outcome (VAS and AOFAS Ankle-Hindfoot Score) using a repeated-measures Friedman test for more than 2 measures and a Wilcoxon signed-rank test with Bonferroni correction to identify where the specific differences lay (Figs. 6, and 7).

## Discussion

Numerous indications for gastrocnemius recession in the literature include non-insertional Achilles tendinopathy, flat feet, metatarsalgia, midfoot pain or arthritis, lateral foot pain, plantar fasciitis, and nerve entrapment. It is also indicated in children with clubfoot, spasticity, or cerebral palsy [6–17, 30–34].

We observed an improvement in the original complaints, mainly Achilles tendinitis, calcifying enthesitis, and plantar fasciitis. However, as our sample was not sufficiently homogeneous or large, we were unable to perform a complete evaluation of these conditions. More studies treating a single condition with this technique should be conducted in the future [19].

The purpose of this study was to evaluate the clinical outcomes for dorsiflexion before and after surgery, as well as postsurgical pain caused by the surgery itself and its impact on patient functionality. The purpose of this study was to evaluate the clinical outcomes of dorsiflexion before and after surgery, as well as the postsurgical pain caused by the surgery itself and its impact on patient functionality. The study exclusively shows the clinical results of the resection of the gastrocnemius, not the impact of the tendon resection on the various pathologies for which the surgical technique is indicated.

The Abbocath needle has been used in ultrasound-guided surgery for specific indications, such as Dupuytren’s disease, plantar fasciitis, and carpal tunnel syndrome [26–28].

The technique presented in this article, which had previously been performed on cadaveric specimens [25], is as effective as all other available approaches. The hook-knife is replaced by a 16G Abbocath needle, which minimizes injury and the risk of infection. Invasiveness is minimal (similar to that of an infiltration), with no need for exsanguination, and anesthesia is local. In addition, the procedure is inexpensive, since it can be performed in the office, without an anesthesiologist or operating room.

In our series, we evaluated the increase in dorsiflexion with an initial resection of the plantaris tendon (15 patients had a clearly visible plantaris tendon). The release of this tendon did not bring about a significant improvement in ankle dorsiflexion. Our results were different from those of Kindred et al. [35], who reported an average dorsiflexion of 9 degrees with isolated release of the plantaris tendon.

We were unable to achieve significant dorsiflexion until we progressed with the transverse resection of the gastrocnemius tendon and reached the area of the intermuscular septum of the soleus. At this point, a significant increase in ankle dorsiflexion was observed.

The improvement in joint range in ankle dorsiflexion we recorded was similar to that reported in previous studies [23].

The range of dorsiflexion improved in the medium term, with no weakness in the calf muscles, as reported by other authors, since with level II gastrocnemius resection there is no risk of uncontrolled lengthening or unstable gait [36]. All the patients had recovered full strength at the third month after surgery.

Complications were minor, mainly mild and superficial hematomas, which resolved uneventfully. Although in our 2016 study, in a series of 25 cases in which the Strayer ultrasound-guided technique with a scalpel hook was performed, in 4 patients there was weakness of propulsion that resolved after 3 months [15]^.^

The risk of infection, poor healing and other complications, although rare in the literature, may be slightly higher with larger incisions than with a completely closed procedure, although more studies and larger series are required. Endoscopic gastrocnemius recession is less invasive and may offer advantages over open procedures in terms of enhanced visualization, smaller incisions, shorter operative time, fewer complications, and less morbidity [20, 37]. Moreover, both open and endoscopic recessions still require epidural anesthesia, lower limb exsanguination, and stitches.

The 2 least invasive surgical procedures described to date are endoscopic surgery and ultrasound-guided surgery. Both approaches are performed using a hook-knife [15, 24, 38]. At the latest follow-up, none of the patients in our study were able to identify the location of the entry portal, while some of the patients in the series based on use of the hook-knife were able to identify a tiny, residual spot.

Ultrasound-guided surgery overcomes the disadvantages of endoscopic surgery, since it does not require exsanguination and is performed with local anesthesia and sedation and minimizes the risk of vascular and nerve damage by enabling direct visualization of the structures involved. This contrasts with endoscopic procedures, where the nerve must be identified after inserting the instruments and camera, thus putting it at risk during the initial stages [15, 24, 39].

An important drawback of our study is that gastrocnemius lengthening is indicated for several foot conditions. Consequently, the limitations are that the sample was not homogeneous, low sample size, short follow-up, and the evaluation of clinical results with respect to foot complaints was limited.

Even more, we have collected the AOSFAS score and presurgical VAS that scored the functional limitation caused by the main pathology, but the results of study are mainly focused on the recovery of the procedure, the gastrocnemius resection. Larger series and homogeneous series are required to carry out a reliable evaluation of the impact and results of the resection of the gastrocnemius on different pathologies for which the surgical technique is indicated.

However, we believe that this does not diminish the benefit of the technique or its effectiveness (as demonstrated by our findings), the eventual improvement in dorsiflexion, and the favorable progress reflected in the medium-term clinical outcomes of the surgical procedure.

## Conclusion

Ultrasound-guided recession of the gastrocnemius tendon with a needle seems to be a precise and safe surgical technique. It minimizes injury and the risk of infection, and invasiveness is similar to that of an infiltration.

In addition, the procedure is relatively inexpensive, since it can be performed in the office under local anesthesia, without the need for an anesthesiologist or operating room.

## Data Availability

Can be requested from the corresponding author.
